# The role of electroconvulsive therapy in the treatment of aggression in psychiatry: A literature Review

**DOI:** 10.1192/j.eurpsy.2025.2131

**Published:** 2025-08-26

**Authors:** A. Rami

**Affiliations:** 1 Razi Hospital, Manouba, Tunisia

## Abstract

**Introduction:**

Electroconvulsive therapy (ECT) has gained increasing attention as a therapeutic option for managing aggressive behavior in psychiatric patients. Aggression is a common symptom in several psychiatric disorders, such as schizophrenia, bipolar disorder, and severe depression, which can be resistant to conventional pharmacological treatments.

**Objectives:**

This literature review examines the efficacy and safety of ECT in reducing aggression across various psychiatric populations.

**Methods:**

We have conducted a web resurch on Pubmed for articles published in the last ten years about the topic using key words like “agression”, “ECT”.

**Results:**

Evidence suggests that ECT can be particularly effective in cases where patients do not respond to medications or display dangerous behaviors. Significant reductions in aggression have been reported post-ECT, along with improvements in mood and overall functioning.

**Conclusions:**

Although ECT remains controversial due to concerns about cognitive side effects, advancements in its application have enhanced its safety. This review emphasizes the need for further.

**Disclosure of Interest:**

None Declared

